# SNP-based breeding for broiler resistance to ascites and evaluation of correlated production traits

**DOI:** 10.1186/s41065-022-00228-x

**Published:** 2022-01-28

**Authors:** Katie Pepper Lee, Nicholas B. Anthony, Sara K. Orlowski, Douglas D. Rhoads

**Affiliations:** 1 Cell and Molecular Biology Program, University of Arkansas, Fayetteville, AR USA; 2 Department of Biological Sciences, University of Arkansas, Fayetteville, AR USA; 3 Department of Poultry Science, University of Arkansas, Fayetteville, AR USA

**Keywords:** Ascites, SNP, Broiler, Breeding

## Abstract

**Background:**

The goal of this study was to evaluate marker-assisted selection (MAS) in broiler chickens using previously mapped gene regions associated with ascites syndrome incidence. The second-generation MAS products were assessed for impact on ascites phenotype and whether there were associated changes in important production traits. Previously, we used whole genome resequencing (WGR) to fine-map 28 chromosomal regions as associated with ascites phenotype in our experimental ascites broiler line (Relaxed, REL) based on a hypobaric chamber challenge. Genotypes for single nucleotide polymorphisms (SNPs) in mapped regions on chromosomes 2 and 22, were used for MAS in our REL line. After two generations, birds homozygous for the genotypes associated with resistance for both chromosomal regions were established. The MAS F_2_ generation was then compared to the REL line for ascites susceptibility and 25 production traits.

**Results:**

Selection based on SNPs in the carboxypeptidase Q (CPQ, Gga2) and leucine rich repeat transmembrane neuronal 4 (LRRTM4, Gga22) gene regions resulted in a sex- and simulated altitude- dependent reduction of ascites incidence in two F_2_ cohorts of the MAS line. Comparisons of the F_2_ MAS and REL lines for production traits when reared at ambient pressure found no significant negative impacts for feed intake (FI), feed conversion ratio (FCR), or deboned part yields for either sex for two F_2_ cohorts. There were, however, improvements in the MAS for full-trial body weight gain (BWG), FCR, absolute and relative tender weights, and relative drumstick weight.

**Conclusions:**

These results validate the mapping of the 28 chromosomal regions and demonstrate that fine mapping by WGR is an effective strategy for addressing a complex trait; it also stands as the first successful SNP-based selection program against a complex disease trait, such as ascites. The MAS line is comparable and, in some instances, superior, in growth performance to the REL control while being more resistant to ascites. This study indicates that MAS based on WGR can provide significant breeding potential in agricultural systems.

**Supplementary Information:**

The online version contains supplementary material available at 10.1186/s41065-022-00228-x.

## Background

Since the 1950s, poultry breeding programs have selected for the increased ability of broilers to rapidly accrete muscle tissue, thus minimizing grow-out time and increasing profits. There have been, however, some negative results of this genetic progress, one of which is pulmonary hypertension syndrome (PHS), or ascites. Traditionally, ascites has been associated with rearing birds at higher elevations where partial pressures of oxygen are lower [1–11], or in colder rearing environments [12, 13]. Ascites syndrome is the terminal result of prolonged pulmonary hypertension, as liver damage releases ascitic fluid into the body cavity [9, 14, 15]. Prolonged hypertension is driven by increased oxygen demands of a rapidly growing body supplied by an inadequate cardiopulmonary system [16–18]. The incidence can be amplified as a result of an oxygen-reduced environment, or increased thermoregulation in cold environments [9, 15]. The bird’s body increases blood pressure in an attempt to respond to metabolic demands, which leads to incomplete gas exchange in the lungs [15, 19–21]. Semi-oxygenated blood is then sent to the organs with substantial detrimental effect on the liver, causing the accumulation of abdominal fluid. It has been estimated that ascites is the cause of up to 8% of broiler mortality and accounts for up to $100,000,000 in economic losses annually, making it both a significant animal welfare and economic concern [6, 14, 15, 21–25].

Mitigation of ascites incidence has achieved varying degrees of success typically employing i) feed restriction [1, 24, 26–31], ii) nutrient density modification to reduce protein [29, 31–33], or iii) feed additives such as arginine or antioxidants [29, 30, 34–37]. Several of the methods for reducing ascites simply slow growth and negatively affect flock production performance. Variability of efficacy found in these mitigation methods can result from genetic differences between commercial lines, environmental variations due to geography, and flock management. As ascites is estimated to have a relatively high heritability with reports ranging from 0.22 to 0.41, it is logical that increased ascites resistance through genetic selection could have significant advantages and increase production potential [4, 11, 38–40].

Previous research at the University of Arkansas on the genetic basis of ascites involved the development of three research lines from a commercial elite line through divergent selection for ascites resistance when exposed to simulated high elevation conditions [11]. The base population (Relaxed, REL) for the selection study was derived from a commercial elite line in the 1990s and maintained through random mating without selection. Sib-selection based on ascites phenotype assessed through a 6-week hypobaric chamber challenge produced ascites resistant (RES) and ascites susceptible (SUS) lines. Rapid response in divergent selection with successive generations suggested a limited number of major genes. A series of genome-wide association studies (GWAS) using SNP panels identified a few candidate SNPs as associated with ascites phenotype, but subsequent MAS-based breeding projects were unsuccessful in validating these few loci [41–44]. More recently, whole genome resequencing (WGR) identified 28 genomic regions where SNP clusters (100s to 1000s of SNPs) showed frequency bias with respect to ascites phenotype [45, 46]. Two of these regions were validated by further genotyping of additional DNA samples and found to have potential epistatic interaction. One region spanned more than 120 kbp on chromosome 2 including the 3′ end of the gene for carboxypeptidase Q (CPQ). The second was an approximately 50 kbp region on chromosome 22 spanning the 3′ end of the gene for leucine-rich repeat transmembrane neuronal 4 (LRRTM4). Both these genes have been associated in human GWAS with blood traits, heart rate, and blood pressure consistent with factors contributing to ascites incidence in poultry.

Therefore, the current study reports on whether MAS based on SNP genotypes for the regions of both CPQ and LRRTM4 can produce offspring with greater innate ascites resistance. Since ascites susceptibility could potentially be linked to important production traits, we also assessed the impact of selection on important broiler production traits.

## Methods

All breeding, hatching, grow-out, and processing took place at the University of Arkansas Poultry Research farm. All animal procedures were approved by the University of Arkansas Institutional Animal Care and Use Committee (Approval Numbers 18083 and 18088) and performed in accordance with relevant guidelines and regulations. This study is reported in accordance with ARRIVE guidelines (https://arriveguidelines.org).

### Bleeding, genotyping, and husbandry of breeder stock

Birds used for breeding were genotyped by collecting 10 μl of blood from the brachial vein which was further processed using a rapid DNA extraction method [47]. These DNAs were then genotyped using exonuclease assays run in triplicate for both CPQ and LRRTM4 genes, as described [45, 46]. Selected breeders were then moved into individual breeder cages for insemination and production of MAS offspring. At 18 weeks of age, the birds were put on a lighting schedule to induce egg production: a starting schedule of 12 h light:12 h dark that progressed for 4 weeks to 16 h light:8 h dark, which was maintained through the insemination/egg collection period. Insemination occurred two times weekly, and eggs were collected daily and were labeled by hen. All eggs were stored at 18 °C and 60% relative humidity until sufficient numbers were reached to begin hatching the next generation.

### Hatchery protocol

Sets of eggs were placed in a setting incubator (Jamesway Incubator Co., Cambridge, Ontario, Canada) at 99.6 °F and 85% relative humidity for 18 days. On d 18, the eggs were candled, infertile eggs removed, fertile eggs placed into hatch baskets, and transferred by mating combination to a hatching incubator (Jamesway Incubator Co.) at 98.0 °F and 88% relative humidity. On d 21, hatched chicks were wing-banded using different band colors for each sub-population, and individual band numbers recorded.

### Hypobaric trials

For both hypobaric cohorts, birds of each line (MAS and REL) were mixed in one of 40 battery cages (measuring 0.6 × 0.6 × 0.3 m) with appropriate numbers from each line to maintain even distribution throughout all the cages. No mortality data other than wing band number was collected for the first week so that final mortality rates would not be confounded by chicks that failed to start. For the remaining 4 weeks of the trial, the husbandry and necropsy methods followed those described previously [11, 41, 44]. As the birds grew, bird densities were reduced in cages to maintain compliance with animal welfare requirements. Birds were initially chosen for removal due to observation of clinical ascites phenotype through palpation of the abdomen. All birds removed were euthanized and subsequently necropsied for ascites phenotype. Once all birds showing ascites phenotype had been removed, additional birds were chosen at random to meet welfare requirements and maintain consistent numbers for both lines. All birds remaining at the end of 5 weeks were euthanized and necropsied to determine ascites phenotype.

### Floor trials

Hatches were placed in floor pens that were top-dressed with fresh pine shavings. Similar numbers for each line were placed at similar densities. Surplus chicks were placed in a separate pen. Cardboard trays for feed were placed and remained in the pens for the first 7 days of the trial. Feed and water were provided ad libitum throughout the trial. One continuous water line per row of pens was adjusted as needed for bird height. Health inspections occurred twice daily at a minimum. All mortalities were removed upon discovery and wing band number, pen of origin, body weight, and any clinical observations including ascites incidence were recorded. If available, the bird was replaced with another of the same sex and genetic line from the surplus birds. Feed was formulated to Cobb-Vantress, Inc., industry recommended standards (formulations can be found in Supplemental Table 1) and was added as needed throughout the trial. Feeding phases were as follows: starter from placement to d 14, finisher from d 14 to d 35, and withdrawal from d 35 to d 55. Pen weights were collected at time of placement, d 14, d 28, d 42, and d 49. Feed intake and feed conversion ratio (FCR) were recorded from d 49 until processing.

### Processing

The day prior to processing, a subset of each cohort consisting of 25 males and 25 females per genetic line (*n* = 100 per cohort) were randomly selected. These birds were removed from the pen first on the morning of processing and were removed from the processing line prior to evisceration to be necropsied for organ weights of liver, lungs (set), spleen, and heart, as well as right ventricle to total ventricular weight (RVTV). These birds were not chilled or deboned. Also on the day prior to processing, a second subset from each cohort were randomly chosen, consisting of 50 males and 50 females per genetic line (*n* = 200 per cohort). These birds were wing-banded to be assessed for MAS impact on meat quality measurements of the breast fillet. Feed was removed 10 h prior to processing to ensure feed passage. All birds were collected the morning of processing and transported to the University of Arkansas Poultry Pilot Processing Plant. At the processing plant, back dock live weight was collected prior to the birds being electrically stunned and exsanguinated, followed by a scalding water bath to loosen feathers, then feather, head, and paw removal. Carcasses were then eviscerated, and the hot carcass and fat pad weights were collected. Carcasses were chilled for 3 h and deboned thereafter for the determination of absolute weight and relative (to back dock live weight) yield of wings, breasts, tenders, thighs, and drumsticks. The 200 carcasses marked for further evaluation were processed as described but also evaluated for muscle quality traits including breast fillet weight, color, and pH at 4- and 24-h chill time. The deviation between the 4- and 24-h fillet weights were used to calculate drip loss. Color and pH were measured using a Minolta CR-400 handheld model with PC-linked SpectramagicX software and Testo model 205 handheld spear-tip probe, respectively. Color readings were taken on the dorsal surface of the breast, while pH was measured in the cranial region of the breast. The breasts were then frozen until the completion of both trials for cooking and shear force measurements. Shear requirements were calculated based on four measurements in the cranial region using a TA.XT*Plus* equipped with a Meullenet-Owens Razor Shear head attachment.

### Statistical methods

All statistical analyses other than the survival model were conducted in R with statistical significance denoted by a *P*-value ≤0.05.

Hypobaric mortality data were analyzed using a generalized linear model (RStudio Team, 2016) of final ascites mortality as well as a survival model which showed the effect of genetic line and sex on the probability of survival over time.

Live performance data were analyzed using two-way ANOVA between the main effects of trial and genetic line. Parts weights, organ weights, heart characteristic, and meat quality characteristic data from processing were analyzed using three-way ANOVA between the main effects of trial, genetic line, and sex. All means were separated by Tukey’s Honest Significant Difference (HSD) test.

## Results

### First-generation breeding

Breeders for the first parent generation were selected from REL breeder stock in July of 2018. After bleeding and genotyping, two P_0_ crosses were then created: one consisting of birds with all non-reference SNPs (compared to galGal6, [46]) for each gene region, designated P_0_–1, and the other consisting of birds with non-reference SNPs for CPQ and heterozygous for SNPs for LRRTM4, designated P_0_–2. P_0_–1 consisted of 10 males and 13 females; P_0_–2 consisted of 12 males and 24 females. Separately, ungenotyped REL birds were used to breed generation 1 of the control population. Semen was collected from all the males of each P_0_ group, pooled, and used to artificially inseminate each of the females from the same P_0_ group.

### Second-generation breeding

After hatching, the F_1_ progeny produced from P_0_–1 and P_0_–2 were kept in floor pens and managed as breeders until they reached 18 weeks of age, at which point they were then bled and genotyped. Breeders for each F1 population were placed in individual breeder cages and photo stimulated. In May of 2019, breeders from the F_1_ were selected based on being homozygous for the non-reference SNPs for both genes; though all breeder SNP genotypes from this point forward were the same, the F1 populations were kept separate in order to complete reciprocal matings between them. Breeders from F_1_–1 (from P_0_–1) consisted of 12 males and 31 females; F_1_–2 (from P_0_–2) consisted of 12 males and 37 females. To produce the control group, 24 males and 72 females from the REL line were also utilized. Insemination began at the same time, when the MAS breeders were 22 weeks of age and the REL control breeders were 20 weeks of age. For the REL, pooled semen from all 24 males were used to artificially inseminate all 72 REL females. For the MAS, pooled semen from the F_1_–1 males was used to artificially inseminate the F_1_–2 females and similarly the F_1_–2 males were used to inseminate the F_1_–1 females. This reciprocal mating scheme produced the F2 generation of birds which would possess only the non-reference SNPs for the CPQ and LRRTM4 genes. Sets of eggs for hatching included at least 250 eggs each from REL and the F_2_ of the MAS. At transfer, all eggs were candled and infertile or eggs with embryonic mortality were removed and stored for breakout on hatch day along with eggs that did not hatch; no significant difference (*P* > 0.05) in hatchery breakout was found between the two lines. After hatch, birds received a wing band that represented their genetic line (either MAS or REL). Sets of eggs were produced every 2 weeks for four total hatches. The first and fourth hatches were subjected to 5-week hypobaric challenges. The second and third hatches were placed for 8-week floor pen trials to evaluate change in production traits associated with MAS.

### Hypobaric challenges

F2 chicks for challenge in the hypobaric (Hypo) chamber were placed on November 28, 2019 (Hypo1) and January 8, 2020 (Hypo2). Hypo1 was maintained at 9000 ft. simulated altitude while Hypo2 was initially set at 9000 ft. simulated altitude, then after 2 weeks increased to 11,000 ft. simulated elevation to induce a higher incidence of ascites. In Hypo1, all hatched birds were placed in the chamber (*n* = 578) whereas in Hypo2, similar numbers of birds were placed from each line and fewer total birds were placed (*n* = 433) which reduced the number of birds that would need to be culled for compliance with bird density regulations.

**Fig. 1:**
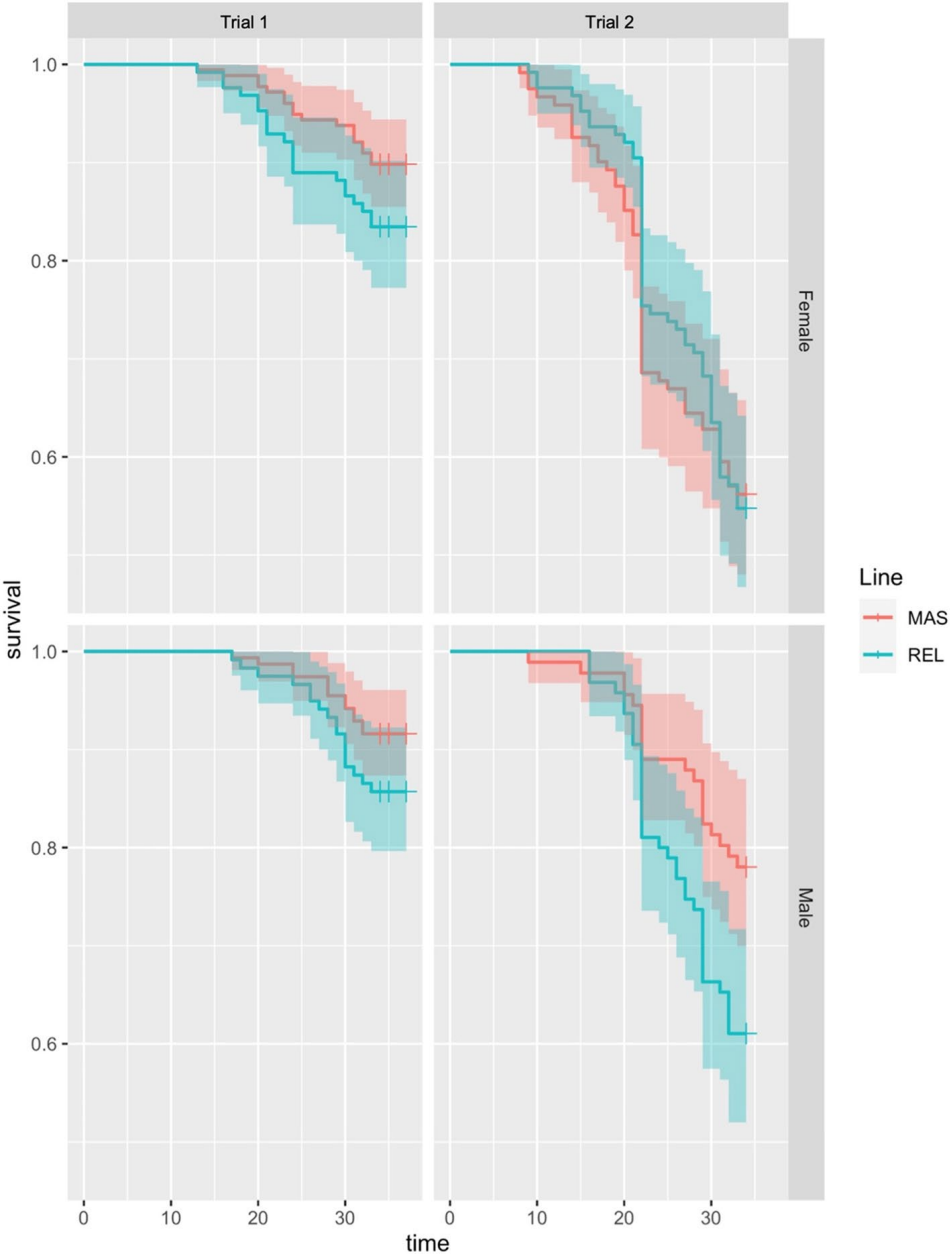
Survival plots for the two hypobaric chamber challenges comparing the MAS and REL lines according to sex

The hypobaric challenge results indicate a sex- and elevation-dependent reduction in ascites incidence in both cohorts. Hypo1 saw an overall decrease (*P* = 0.041) in ascites mortality between the MAS and REL birds, with a 27.3% reduction for ascites in MAS males and a 39.8% in MAS females [Table 1]. For Hypo2, there was an overall numerical, although not statistically significant (*P* = 0.162), decrease in ascites mortality between the lines, with reductions of 23.4% in males (*P* = 0.126) and only 5.2% reduction in females [Table 2]. There was no significant difference (*P* > 0.05) between the lines for the right ventricle to total ventricle (RVTV) ratio or body weight. The Kaplan-Meier survival model curve visually echoes these trends, however the analyzed *P*-values from this model are only numerically different (P > 0.05) [Fig. 1].

**Table 1:** Ascites incidence and cardiac hypertrophy (RVTV) for the two hypobaric trials comparing the MAS and REL lines overall and by gender

Item	n	RVTV	Ascites			
			No	Yes	Percent, %	Difference, %
**Hypobaric trial 1**						
Sex						
Male	274	0.305	237	37	13.50	28.00
Female	304	0.321	247	57	18.75	
Line						
MAS	332	0.310	288	44	13.25	34.83
REL	246	0.318	196	50	20.33	
Interactions						
Male × MAS	155	0.299	137	18	11.61	27.27
Male × REL	119	0.314	100	19	15.97	
Female × MAS	177	0.319	151	26	14.69	39.82
Female × REL	127	0.323	96	31	24.41	
*P*-value						
Sex		0.009			0.047	
Genetic Line		0.160			0.041	
Sex × Line		0.367			0.742	
**Hypobaric Trial 2**						
Sex						
Male	186	0.387	108	78	41.94	30.47
Female	247	0.421	98	149	60.32	
Line						
MAS	212	0.403	108	104	49.06	11.86
REL	221	0.410	98	123	55.66	
Interactions						
Male × MAS	91	0.39	58	33	36.26	23.44
Male × REL	95	0.39	50	45	47.37	
Female × MAS	121	0.42	50	71	58.68	5.21
Female × REL	126	0.43	48	78	61.90	
*P*-value						
Sex		< 0.001			< 0.001	
Genetic Line		0.404			0.162	
Sex × Line		0.550			0.415

**Table 2:** Live performance data from the replicate floor trials

Item	n								d49–54	
		d0, g/bird	d14, g/bird	d28, g/bird	d42, g/bird	d49, g/bird	d54, g/bird	BWG, g/bird	FI, g/bird	FCR, g:g
**Main effect of trial**										
Floor1	40	38.00^a^	255.0^a^	934.8	1987^a^	2522^a^	2888^a^	365.5	0.932	2.566
Floor2	40	36.65^b^	236.7^b^	947.7	1949^b^	2458^b^	2822^b^	368.3	0.937	2.560
SEM		0.15	2.1	6.3	13	16	20	4.6	0.007	0.021
**Main effect of genetic line**										
MAS	40	37.30	246.4	940.3	1976	2507	2875	372.9^a^	0.935	2.512^b^
REL	40	37.35	245.3	942.2	1960	2474	2835	361.0^b^	0.933	2.613^a^
SEM		0.19	2.8	6.4	13	17	20	4.3	0.007	0.021
**Trial × Line**										
Floor1 × MAS	20	38.06	252.4^a^	927.9	1993	2541	2909	368.4	0.928	2.523
Floor1 × REL	20	37.94	257.6^a^	941.8	1981	2504	2867	362.5	0.935	2.609
Floor2 × MAS	20	36.54	240.4^b^	952.6	1960	2473	2842	377.7	0.943	2.500
Floor2 × REL	20	36.76	233.0^b^	942.7	1939	2444	2803	359.5	0.931	2.618
SEM		0.22	3.2	9.7	21	26	31	7.1	0.010	0.031
***P*-values**										
Trial		**< 0.001**	**< 0.001**	0.142	**0.038**	**0.006**	**0.017**	0.585	0.583	0.773
Genetic line		0.800	0.674	0.819	0.358	0.155	0.137	**0.036**	0.812	**< 0.001**
Trial × Line		0.393	**0.019**	0.174	0.801	0.872	0.946	0.279	0.303	0.536

*Abbreviations*: *Floor1* Floor cohort 1, *Floor2* Floor cohort 2, *MAS* marker-assisted selection line, *REL* Relaxed (control) line, *d* day, *BWG* body weight gain, *FI* feed intake, *FCR* feed conversion ratio

Item values with different superscript letters in a column indicates significant difference (*p* < 0.05) in that trait or effect

### Floor trials

Birds reared for the floor trials were placed on December 12, 2019 (Floor1) and December 28, 2019 (Floor2). On the day of placement, the total number of hatched birds was counted for each line and the number of birds placed per pen determined based on the smallest group; for Floor1 this was 14 birds per pen (0.133 m^2^/bird) and for Floor2 13 birds per pen (0.143 m^2^/bird) with all excess birds placed in spare pens for mortality replacement up until the beginning of the FCR measurement period from d 49 to d 55. As both floor trials were run concurrently in the same barn, though offset by 2 weeks, we considered whether the live performance and processing data should be analyzed as completely separate trials with separate analyses or together by adding the main effect of trial. After consultation with a professional agricultural statistician and colleagues at the University of Arkansas, the latter option was chosen. However, the interaction between line and trial was assessed for each measurement, and any traits identified as having an interaction between line and trial were noted and are discussed. Conclusions about the overall affect of MAS on that particular measurement were not drawn in those cases.

Live performance data from both cohorts is shown in Table 2. Significant differences were found between trials: d0 body weight (BW) (*P* < 0.001), d42 BW (*P* = 0.038), d0–42 body weight gain (BWG) (*P* = 0.045), d49 BW (*P* = 0.006), d0–49 BWG (*P* = 0.007), d54 BW (*P* = 0.017), and full-trial BWG (*P* = 0.019). Additionally, the genetic lines were significantly different in d49–54 BWG (*P* = 0.036) and FCR (P < 0.001), both of which were improved in the MAS. Significant differences were found between trial and genetic line for d14 BW (P = 0.019) and d0–14 BWG (*P* = 0.015).

### Processing

Total sample sizes and tabular representations of the data are as follows: Table 3, live weight and carcass characteristics (*n* = 868); Table 4, deboned parts (n = 868); Table 5, organ weights (*n* = 295); Table 6, heart characteristics (n = 295); Table 7, meat quality characteristics (*n* = 395). A significant improvement was seen in the MAS birds for absolute and relative tender weights (*P* < 0.001, *P* < 0.001, respectively), relative drumstick weight (*P* = 0.016), and significant differences were found for 24-h L* color measurement (*P* < 0.001), 24-h a* color measurement (*P* = 0.037), and 24-h pH (*P* = 0.003). Significant differences were found between sexes in relative hot carcass weight (*P* < 0.001), wing weight (*P* < 0.001), relative thigh weight (P < 0.001), relative drumstick weight (*P* < 0.001), absolute and relative heart weight (P < 0.001, *P* = 0.018, respectively), liver weight (P < 0.001), lung weight (P < 0.001), spleen weight (*P* < 0.001), RV weight (P < 0.001), TV weight (P < 0.001), drip loss (P < 0.001), 24-h L* color measurement (P < 0.001), 24-h pH (P < 0.001), and shearing peaks (*P* = 0.016).

**Table 3:** Live weight and carcass characteristics from the replicate floor trials

Item	n	Live	Hot carcass		Fat pad		Chilled carcass	
		Weight, kg	Weight, kg	Yield^1^, %	Weight, kg	Yield, %	Weight, kg	Yield, %
**Main effect of trial**								
Floor1	453	2814^a^	1996^a^	70.94	67.20^a^	2.402^a^	2058^a^	73.13
Floor2	415	2730^b^	1941^b^	71.11	59.53^b^	2.194^b^	2002^b^	73.35
SEM		19	14	0.07	0.88	0.030	14	0.07
**Main effect of genetic line**								
MAS	439	2795^a^	1988^a^	71.12	64.03	2.307	2050^a^	73.33
REL	429	2752^b^	1951^b^	70.91	63.00	2.297	2013^b^	73.14
SEM		19	14	0.07	0.91	0.031	14	0.07
**Main effect of sex**								
Male	395	3117^a^	2223^a^	71.33^a^	65.56^a^	2.104^a^	2281^a^	73.22
Female	473	2488^b^	1759^b^	70.75^b^	61.81^b^	2.469^b^	1823^b^	73.25
SEM		13	10	0.07	0.91	0.029	10	0.08
**Trial × Line**								
Floor1 × MAS	233	2826	2010	71.09	67.00	2.385	2072	73.29
Floor1 × REL	220	2803	1983	70.77	67.42	2.420	2045	72.97
Floor2 × MAS	206	2761	1964	71.15	60.67	2.218	2025	73.38
Floor2 × REL	209	2699	1918	71.06	58.41	2.170	1979	73.33
SEM		27	20	0.10	1.36	0.045	20	0.11
**Trial × Sex**								
Floor1 × Male	204	3174^a^	2264^a^	71.29	70.71^a^	2.236	2322^a^	73.13
Floor1 × Female	249	2513^c^	1773^c^	70.65	64.31^b^	2.540	1838^c^	73.14
Floor2 × Male	191	3055^b^	2180^b^	71.38	60.07^c^	1.962	2238^b^	73.32
Floor2 × Female	224	2460^c^	1743^c^	70.87	59.08^bc^	2.391	1806^c^	73.38
SEM		19	15	0.10	1.29	0.042	15	0.11
**Line × Sex**								
MAS × Male	209	3147^a^	2248^a^	71.42	66.98	2.128	2306^a^	73.30
MAS × Female	230	2476^c^	1753^c^	70.84	61.33	2.469	1818^c^	73.36
REL × Male	186	3085^b^	2197^b^	71.23	63.95	2.076	2255^b^	73.13
REL × Female	243	2498^c^	1764^c^	70.67	62.27	2.468	1827^c^	73.15
SEM		21	16	0.10	1.38	0.042	16	0.11
**Trial × Line × Sex**								
Floor1 x MAS x Male	114					2.201^cd^		
Floor1 x MAS x Female	118					2.564^a^		
Floor1 x REL x Male	90					2.281^bcd^		
Floor1 x REL x Female	127					2.518^ab^		
Floor2 x MAS x Male	95					2.042^de^		
Floor2 x MAS x Female	111					2.369^abc^		
Floor2 x REL x Male	96					1.883^e^		
Floor2 x REL x Female	113					2.413^abc^		
SEM						0.062		
***P*-values**								
Trial		**< 0.001**	**< 0.001**	0.078	**< 0.001**	**< 0.001**	**< 0.001**	0.270
Genetic line		**0.008**	**0.003**	0.054	0.313	0.618	**0.003**	0.069
Sex		**< 0.001**	**< 0.001**	**< 0.001**	**0.002**	**< 0.001**	**< 0.001**	0.640
Trial × Line		0.235	0.432	0.293	0.274	0.349	0.468	0.174
Trial × Sex		**0.035**	**0.028**	0.514	**0.019**	0.108	**0.036**	0.909
Line × Sex		**0.011**	**0.012**	0.942	0.200	0.628	**0.019**	0.915
Trial × Line × Sex		0.980	0.992	0.637	0.093	**0.039**	0.963	0.889

*Abbreviations*: *Floor1* Floor cohort 1, *Floor2* Floor cohort 2, *MAS* marker-assisted selection line, *REL* Relaxed (control) line

1: Yields calculated based on back-dock live weight

Item values with different superscript letters in a column indicate significant difference (*p* < 0.05) in that trait or effect

**Table 4:** Deboned parts characteristics from the replicate floor trials

Item	n	Wings		Breast fillets		Tenders		Thighs		Drumsticks	
		Weight, kg	Yield^1^, %	Weight, kg	Yield, %	Weight, kg	Yield, %	Weight, kg	Yield, %	Weight, kg	Yield, %
**Main effect of trial**											
Floor1	453	223.2^a^	7.947	431.6	15.33^b^	112.2	4.001^b^	380.4^a^	13.47^a^	271.9^a^	9.653^a^
Floor2	415	216.6^b^	7.936	432.4	15.79^a^	111.2	4.080^a^	362.7^b^	13.25^b^	260.3^b^	9.537^b^
SEM		1.5	0.020	3.7	0.07	0.8	0.020	3.3	0.05	2.1	0.027
**Main effect of genetic line**											
MAS	439	221.2	7.928	435.9	15.57	114.0^a^	4.091^a^	376.3^a^	13.41	269.8^a^	9.649^a^
REL	429	218.9	7.956	427.9	15.54	109.5^b^	3.985^b^	367.5^b^	13.31	262.9^b^	9.546^b^
SEM		1.5	0.020	3.7	0.07	0.8	0.018	3.3	0.05	2.1	0.027
**Main effect of sex**											
Male	395	247.5^a^	7.943	483.2^a^	15.49	121.8^a^	3.907^b^	427.6^a^	13.71^a^	302.7^a^	9.723^a^
Female	473	197.2^b^	7.940	389.1^b^	15.60	103.3^b^	4.149^a^	325.4^b^	13.07^b^	236.2^b^	9.494^b^
SEM		1.0	0.019	3.2	0.07	0.7	0.018	2.5	0.05	1.5	0.027
**Trial × Line**											
Floor1 × MAS	233	224.1	7.925	436.7	15.37	114.4	4.057	383.5	13.53	274.7	9.717
Floor1 × REL	220	222.2	7.970	426.1	15.29	110.0	3.941	377.4	13.39	269.1	9.586
Floor2 × MAS	206	217.9	7.932	435.0	15.79	113.5	4.130	368.2	13.27	264.1	9.571
Floor2 × REL	209	215.4	7.940	429.8	15.80	108.9	4.031	357.1	13.22	256.5	9.504
SEM		2.2	0.029	5.3	0.10	1.2	0.029	4.7	0.08	3.0	0.040
**Trial × Sex**											
Floor1 × Male	204	252.1	7.938	486.7	15.34	123.6^a^	3.899^c^	439.6^a^	13.85	310.2^a^	9.779
Floor1 × Female	249	199.4	7.954	386.2	15.33	102.7^c^	4.084^b^	330.9^c^	13.15	240.0^c^	9.550
Floor2 × Male	191	242.5	7.949	479.4	15.66	119.9^b^	3.915^c^	414.8^b^	13.57	294.7^b^	9.662
Floor2 × Female	224	194.7	7.925	392.3	15.90	104.0^c^	4.220^a^	319.3^d^	12.97	231.9^d^	9.433
SEM		1.3	0.029	4.7	0.10	1.1	0.027	3.6	0.08	2.0	0.038
**Line × Sex**											
MAS × Male	209	248.8	7.911	489.8^a^	15.53	124.2	3.944	434.2^a^	13.79	306.8^a^	9.765
MAS × Female	230	196.3	7.944	387.0^b^	15.60	104.7	4.225	323.7^c^	13.07	236.4^c^	9.544
REL × Male	186	246.0	7.980	475.8^a^	15.45	119.4	3.865	420.6^b^	13.63	298.5^b^	9.675
REL × Female	243	198.0	7.937	391.0^b^	15.61	101.8	4.076	326.7^c^	13.06	235.7^c^	9.447
SEM		1.7	0.028	5.0	0.11	1.1	0.026	3.7	0.08	2.3	0.041
***P*-values**											
Trial		**< 0.001**	0.630	0.834	**< 0.001**	0.296	**< 0.001**	**< 0.001**	**< 0.001**	**< 0.001**	**0.002**
Genetic line		0.840	0.286	0.324	0.665	**< 0.001**	**< 0.001**	**0.003**	0.258	**< 0.001**	**0.016**
Sex		**< 0.001**	0.773	**< 0.001**	0.180	**< 0.001**	**< 0.001**	**< 0.001**	**< 0.001**	**< 0.001**	**< 0.001**
Trial × Line		0.085	0.393	0.698	0.532	0.924	0.631	0.404	0.897	0.564	0.563
Trial × Sex		0.053	0.558	0.079	0.210	**0.010**	**0.014**	**0.025**	0.481	**0.032**	0.903
Line × Sex		0.213	0.185	**0.050**	0.538	0.339	0.127	**0.006**	0.287	**0.032**	0.842
Trial × Line × Sex		0.245	0.073	0.568	0.441	0.725	0.668	0.607	0.251	0.906	0.827

*Abbreviations*: *Floor1* Floor cohort 1, *Floor2* Floor cohort 2, *MAS* marker-assisted selection line, *REL* Relaxed (control) line

1: Yields calculated based on back-dock live weight

Item values with different superscript letters in a column indicate significant difference (*p* < 0.05) in that trait or effect

**Table 5:** Organ weights from the replicate floor trials

Item	n	Heart		Liver		Lungs		Spleen	
		Weight, g	Yield^1^, %	Weight, g	Yield, %	Weight, g	Yield, %	Weight, g	Yield, %
**Main effect of trial**									
Floor1	95	14.95	0.626^b^	50.91^a^	2.108	15.78	0.663	2.878	0.122^b^
Floor2	100	15.17	0.666^a^	47.51^b^	2.077	15.47	0.680	3.083	0.135^a^
SEM		0.30	0.009	1.05	0.034	0.36	0.013	0.079	0.003
**Main effect of genetic line**									
MAS	98	15.07	0.643	49.78	2.108	15.43	0.663	3.001	0.129
REL	97	15.06	0.651	48.58	2.076	15.82	0.681	2.965	0.128
SEM		0.31	0.009	0.98	0.030	0.37	0.013	0.080	0.003
**Main effect of sex**									
Male	98	17.01^a^	0.660^a^	51.42^a^	1.983^b^	17.16^a^	0.664	3.176^a^	0.123
Female	97	13.10^b^	0.633^b^	46.88^b^	2.208^a^	14.09^b^	0.680	2.788^b^	0.134
SEM		0.20	0.008	1.05	0.032	0.34	0.013	0.083	0.003
**Trial × Line**									
Floor1 × MAS	48	15.02	0.624	51.40	2.111	15.78	0.661	2.808	0.118
Floor1 × REL	47	14.88	0.629	50.43	2.105	15.78	0.665	2.951	0.125
Floor2 × MAS	50	15.11	0.660	48.18	2.106	15.09	0.664	3.186	0.139
Floor2 × REL	50	15.24	0.671	46.84	2.048	15.85	0.696	2.978	0.130
SEM		0.49	0.013	1.57	0.050	0.57	0.019	0.114	0.004
**Trial × Sex**									
Floor1 × Male	48	17.00	0.638	52.54	1.960^b^	17.51	0.655	3.098	0.116
Floor1 × Female	47	12.88	0.615	49.26	2.269^a^	14.05	0.671	2.655	0.127
Floor2 × Male	50	17.02	0.681	50.34	2.006^b^	16.81	0.672	3.250	0.129
Floor2 × Female	50	13.31	0.650	44.61	2.151^a^	14.13	0.689	2.914	0.140
SEM		0.31	0.013	1.72	0.050	0.57	0.022	0.122	0.004
**Line × Sex**									
MAS × Male	48	17.19	0.663	52.28	1.999	17.01	0.654	3.231	0.124
MAS × Female	50	13.02	0.623	47.33	2.220	13.95	0.670	2.780	0.134
REL × Male	50	16.83	0.657	50.58	1.969	17.31	0.672	3.122	0.122
REL × Female	47	13.19	0.644	46.45	2.194	14.22	0.690	2.798	0.134
SEM		0.32	0.012	1.50	0.049	0.50	0.019	0.133	0.005
***P*-values**									
Trial		0.434	**< 0.001**	**0.009**	0.348	0.470	0.332	0.052	**0.014**
Genetic Line		0.999	0.466	0.357	0.508	0.370	0.283	0.731	0.446
Sex		**< 0.001**	**0.018**	**< 0.001**	**< 0.001**	**< 0.001**	0.322	**< 0.001**	0.556
Trial × Line		0.646	0.707	0.888	0.396	0.384	0.447	0.116	0.130
Trial × Sex		0.469	0.690	0.347	**0.032**	0.364	0.964	0.636	0.610
Line × Sex		0.363	0.284	0.752	0.948	0.971	0.986	0.580	0.978
Trial × Line × Sex		0.284	0.396	0.601	0.660	0.396	0.692	0.806	0.652

*Abbreviations*: *Floor1* Floor cohort 1, *Floor2* Floor cohort 2, *MAS* marker-assisted selection line, *REL* Relaxed (control) line

1: Yields calculated based on back-dock live weight

Item values with different superscript letters in a column indicates significant difference (*p* < 0.05) in that trait or effect

**Table 6:** Heart characteristics from the replicate floor trials

Item	n	RV (g)	TV (g)	RV:TV
**Main effect of trial**				
Floor1	95	2.306	10.31	0.225^a^
Floor2	100	2.270	10.43	0.217^b^
SEM		0.056	0.22	0.003
**Main effect of genetic line**				
MAS	98	2.284	10.40	0.220
REL	97	2.292	10.34	0.222
SEM		0.059	0.22	0.003
**Main effect of sex**				
Male	98	2.589^a^	11.85^a^	0.219
Female	97	1.986^b^	8.87^b^	0.223
SEM		0.044	0.14	0.003
**Trial × Line**				
Floor1 × MAS	48	2.354^a^	10.41	0.230^a^
Floor1 × REL	47	2.259^a^	10.20	0.220^ab^
Floor2 × MAS	50	2.218^a^	10.40	0.210^a^
Floor2 × REL	50	2.323^a^	10.46	0.224^ab^
SEM		0.091	0.36	0.005
**Trial × Sex**				
Floor1 × Male	48	2.618	11.96	0.221
Floor1 × Female	47	1.995	8.64	0.230
Floor2 × Male	50	2.562	11.76	0.218
Floor2 × Female	50	1.979	9.09	0.216
SEM		0.066	0.21	0.004
**Line × Sex**				
MAS × Male	48	2.609	12.04	0.218
MAS × Female	50	1.978	8.83	0.221
REL × Male	50	2.571	11.68	0.220
REL × Female	47	1.995	8.91	0.224
SEM		0.075	0.24	0.005
***P*-values**				
Trial		0.538	0.499	**0.049**
Genetic Line		0.802	0.712	0.517
Sex		**< 0.001**	**< 0.001**	0.481
Trial × Line		**0.046**	0.489	**0.005**
Trial × Sex		0.691	0.078	0.192
Line × Sex		0.630	0.237	0.862
Trial × Line × Sex		0.907	0.154	0.075

*Abbreviations*: *Floor1* Floor cohort 1, *Floor2* Floor cohort 2, *MAS* marker-assisted selection line, *REL* Relaxed (control) line, *RV* right ventricle, *TV* total ventricle

Item values with different superscript letters in a column indicates significant difference (*p* < 0.05) in that trait or effect

**Table 7:** Meat quality characteristics from the replicate floor trials

Item	n		24-h Color				Shear		
		Drip loss^1^, g	L*(D65)	a*(D65)	b*(D65)	24-h pH	Force (N)	Area 1:3	Peaks
**Main effect of trial**									
Floor1	198	2.052	52.34	2.050^a^	8.932^a^	5.946^a^	14.04	187.7	8.823^b^
Floor2	197	2.165	52.72	1.776^b^	8.607^b^	5.857^b^	14.26	191.9	9.545^a^
SEM		0.116	0.19	0.071	0.108	0.013	0.23	2.7	0.182
**Main effect of genetic line**									
MAS	196	2.161	53.03^a^	1.805^b^	8.956^a^	5.875^b^	13.85^b^	184.0^b^	8.981
REL	199	2.047	52.04^b^	2.011^a^	8.582^b^	5.926^a^	14.45^a^	195.6^a^	9.384
SEM		0.114	0.19	0.069	0.107	0.013	0.22	2.6	0.179
**Main effect of sex**									
Male	198	1.612^b^	51.50^b^	1.923	8.384^b^	5.935^a^	13.76^b^	186.0^b^	8.859^b^
Female	197	2.617^a^	53.56^a^	1.893	9.157^a^	5.866^b^	14.55^a^	193.7^a^	9.512^a^
SEM		0.125	0.18	0.071	0.106	0.013	0.22	2.7	0.187
**Trial × Line**									
Floor1 × MAS	97	2.117	52.89	1.905	9.093	5.925	14.32^ab^	189.1^b^	8.521
Floor1 × REL	101	1.990	51.79	2.190	8.774	5.966	13.78^b^	186.5^b^	9.113
Floor2 × MAS	99	2.204	53.16	1.706	8.821	5.825	13.39^b^	179.0^b^	9.427
Floor2 × REL	98	2.106	52.30	1.824	8.389	5.885	15.14^a^	204.9^a^	9.667
SEM		0.166	0.27	0.103	0.155	0.018	0.33	3.9	0.268
**Trial × Sex**									
Floor1 × Male	101	1.460	51.12	2.152	8.469	5.980	13.28^b^	180.4^b^	8.441
Floor1 × Female	97	2.696	53.56	1.945	9.414	5.910	14.83^a^	195.3^a^	9.229
Floor2 × Male	97	1.771	51.89	1.682	8.295	5.888	14.26^ab^	191.9^ab^	9.299
Floor2 × Female	100	2.542	53.56	1.842	8.908	5.822	14.28^ab^	192.1^ab^	9.783
SEM		0.179	0.27	0.113	0.149	0.019	0.33	3.9	0.290
**Line × Sex**									
MAS × Male	98	1.670	51.80	1.855	8.411^b^	5.916	13.62	180.7	8.699
MAS × Female	98	2.663	54.25	1.754	9.506^a^	5.833	14.09	187.3	9.265
REL × Male	100	1.556	51.20	1.992	8.356^b^	5.954	13.91	191.3	9.018
REL × Female	99	2.570	52.88	2.029	8.811^b^	5.898	15.01	200.0	9.758
SEM		0.178	0.26	0.104	0.156	0.019	0.32	4.0	0.264
***P*-values**									
Trial		0.629	0.113	**0.003**	**0.017**	**< 0.001**	0.457	0.239	**0.002**
Genetic Line		0.515	**< 0.001**	**0.037**	**0.009**	**0.003**	**0.045**	**0.001**	0.060
Sex		**< 0.001**	**< 0.001**	0.803	**< 0.001**	**< 0.001**	**0.009**	**0.031**	**0.016**
Trial × Line		0.908	0.611	0.367	0.757	0.622	**< 0.001**	**< 0.001**	0.378
Trial × Sex		0.128	0.112	0.052	0.236	0.848	**0.010**	**0.040**	0.681
Line × Sex		0.967	0.112	0.456	**0.025**	0.473	0.287	0.778	0.555
Trial × Line × Sex		0.096	0.794	0.290	0.694	0.766	0.803	0.956	0.667

*Abbreviations*: *Floor1* Floor cohort 1, *Floor2* Floor cohort 2, *MAS* marker-assisted selection line, *REL* Relaxed (control) line

1: Drip loss calculated as the difference in breast weight before and after 24 h chill

Item values with different superscript letters in a column indicates significant difference (*p* < 0.05) in that trait or effect

Significant differences were also found between the two floor trials for many characteristics, including wing weight (*P* < 0.001), relative breast weight (P < 0.001), relative thigh weight (P < 0.001), relative drumstick weight (*P* = 0.002), relative heart weight (P < 0.001), liver weight (*P* = 0.009), relative spleen weight (*P* = 0.014), 24-h a* color measurement (*P* = 0.003), 24-h b* color measurement (*P* = 0.017), 24-h pH (P < 0.001), and shearing peaks (P = 0.002). Due to this, there were also several significant interactions. Between trial and genetic line, significant differences were found for RV weight (*P* = 0.046), RVTV (*P* = 0.005), shear force requirement (P < 0.001), and 1:3 shear area (P < 0.001). While RV was found to be significantly different, Tukey’s HSD test was unable to separate the means. RVTV was found to be the largest in MAS birds from both trials and the smallest in REL birds from both trials. The largest shear force measurements were found in the Floor2 REL breasts, with intermediate force requirements in the Floor1 MAS group, and the lowest requirements in the Floor1 REL and Floor2 MAS groups. The largest 1:3 shear area measurements were found in the Floor2 REL group, with all other groups having comparable lower measurements.

A number of significant differences were found between trial and sex. These were live weight (*P* = 0.035), hot carcass weight (*P* = 0.028), fat pad weight (*P* = 0.019), chilled carcass weight (*P* = 0.036), absolute and relative tender weight (*P* = 0.010, *P* = 0.014, respectively), thigh weight (*P* = 0.025), drumstick weight (*P* = 0.032), relative liver weight (P = 0.032), shear force requirement (P = 0.010), and 1:3 shear area (*P* = 0.040). The live weight and weights of hot carcass, chilled carcass, and tenders were the largest for the Floor1 males, moderate for Floor2 males, and smallest for females in both trials. The largest fat pads were found in the Floor1 males, moderate for Floor1 females, small intermediate for Floor2 females, and smallest in Floor2 males. Relative tender weight was found to be the greatest in Floor2 females, moderate in Floor1 females, and the lowest in males from both trials. The mean weights of thighs and drumsticks were separated into four distinct groups, from largest to smallest being Floor1 males, Floor2 males, Floor1 females, and Floor2 females. The relative weight of liver was found to be the largest in females from both trials and the smallest in males from both trials. For both the shear force requirement and the 1:3 shear area, the measurements from Floor1 females were found to be the largest, the Floor1 males were found to be the smallest, and both sexes in Floor2 were intermediate.

Finally, there were interactions found between genetic line and sex. These were live weight (*P* = 0.011) hot carcass weight (*P* = 0.012), chilled carcass weight (*P* = 0.019), breast weight (*P* = 0.050), thigh weight (*P* = 0.006), drumstick weight (*P* = 0.032), and 24-h b* color measurement (*P* = 0.025). For live weight and the weights of hot carcass, chilled carcass, thighs, and drumsticks, MAS males were found to be the largest, REL males were intermediate, and females from both the MAS and REL lines were found to be the smallest. Breast weight was the largest for males from both the MAS and REL lines, with the smallest weight in the females from both lines. The 24-h b* color measurement was found to be the highest in MAS females, with the three other groups having comparable lower b* measurements.

There was also a single three-way interaction (*P* = 0.039) found for the processing measurements, which was fat pad. In order from largest to smallest, the means were separated as follows: Floor1 MAS females, Floor1 REL females, Floor2 females from both lines, Floor1 REL males, Floor1 MAS males, Floor2 MAS males, and Floor2 REL males.

## Discussion

Ascites has been reported to have a significant sex-dependent incidence in flocks [39, 48]. In the REL line, females show an earlier onset and higher overall incidence of ascites [11]. However, a reduction of ascites incidence was observed between MAS females of the first and fourth cohorts for hypobaric challenges where the simulated altitude differed. At moderate elevations (9000 ft) in Hypo1, ascites incidence was reduced by nearly 40% for the MAS females, while at higher simulated altitude (11,000 ft) in Hypo2, there was only a 5% reduction of female mortality from 61.9% in the REL line to 58.7% in the MAS line. For males, incidence was reduced in the MAS line by 27% in Hypo1, while being reduced by 23% in Hypo2 with the increased simulated altitude. This further demonstrates the sex-linked nature of ascites resistance or susceptibility and suggests more research into epistasis with genes on the sex chromosomes are warranted. Additional research into the impact of MAS on each sex is also warranted. The timeline of this study, and the capacity of the hypobaric chamber, did not allow for single-sex experiments to allow for greater sample sizes. However, even with relatively small cohort sizes, significant reduction of ascites for the MAS line was observed.

An additional point of success in these results comes from the lack of impact on production traits. If MAS negatively impacted economically important traits in any major way, then MAS would be much less appealing to the industry. From the live production data, a significant improvement of the MAS was found in body weight gain from the full trial and in FCR. A limited number of traits showed significant differences between overall MAS and REL averages, all of which were improved in the MAS birds; these were absolute and relative tender weights and relative drumstick weight. Additionally, there were some improvements in the MAS that were only seen in one sex. These were live weight, hot carcass, chilled carcass, thigh weight, and drumstick weight, all of which were larger in MAS males over REL males while the MAS and REL female measurements in each of these cases were statistically equivalent. Given these improvements, MAS appears to be not only capable of reducing ascites incidence, but to have a positive impact on some growth characteristics. Notably, there was a significant three-way interaction between trial, genetic line, and sex for fat pad weight relative to back-dock live weight. From the trend in this characteristic, we observed that the females from each cohort had larger relative fat pad weights than the males, but as this did not have an impact on the trends of the deboned parts data, we are less concerned about this outcome.

We also identified differences between MAS and REL for breast color measurements. The measurement of each breast fillet was broken into three components: L* represents the lightness from 0 (black) to 100 (white); a* represents the color spectrum from − 60 (green) to + 60 (red); b* represents the color spectrum from − 60 (blue) to + 60 (yellow) (American Meat Science Association, 2012). A significant difference was found in the L* and a* measurements between the two genetic lines, and in the L* measurement between the sexes. The difference in L* measurements between the two genetic lines is especially interesting as this means that MAS line breast fillets were consistently lighter in color than the REL. While the classification of Qiao et al. [49] considers all of the L* measurements from both cohorts except for MAS female to be “normal” in lightness (MAS female would be classified as “lighter than normal”). This color difference is difficult to visually discern, however fillets with higher L* values may have higher moisture contents. Thus, the fillets from MAS may have consistently greater moisture than the REL. The data also showed an interaction between genetic line and sex with the b* measurement. However, other changes occurred that were only numeric; some measurements increased nearly one unit between the two cohorts, others changed which group had a greater value for a particular measurement. These variations could trace back to minor differences in formulation of individual batches of feed used for the two trials or to other causes still unknown. While these are relatively small fluctuations, it is difficult to know exactly how much of an impact it might have on consumer perspective of the color of these fillets [50].

These data validate the WGR approach for identifying regions for MAS for improving multigene traits in commercial breeding programs. However, there are limitations. One limitation is that the regions we identified in REL by WGR may not be relevant in current elite lines. The REL is the unselected descendant from a commercial elite line from the 1990s [11]. The genetics of modern elite broiler lines have undoubtedly changed in the ensuing two decades. WGR for ascites in two current commercial broiler crosses did not find associations with either the CPQ or LRRTM4 regions (unpublished). Current elite lines could be subjected to WGR using the hypobaric chamber challenge to identify line-specific regions associated with ascites phenotype. Despite the unique and proprietary nature of each commercial line, each could be assessed individually, candidate gene regions validated, and then informative regions included in selection programs. Alternatively, commercial broilers could be subjected to WGR to identify the regions to be selected for in the elite lines to produce the required genetics in the terminal cross for production of those broiler products. Further research and MAS projects for the major modern commercial crosses are needed to fully understand the efficacy of this method against ascites.

Though its prevalence in the US market varies by specific commercial products (unpublished data) and geographical region, ascites still remains problematic in the global market given worldwide variation in climates, elevations, and management strategies. Published data for mortality and economic impact are nearing or surpass two decades old [21, 25], warranting new assessments of the impact on the US and global markets. Based on existing statistics, significant reduction could potentially prevent millions of bird deaths, saving millions of dollars for the industry. Our results document that genetics can be used to significantly reduce ascites without compromising production.

## Conclusions

These results represent the first documented success in fine-mapping and marker-assisted selection for a complex trait in a poultry species. WGR has the potential to not only identify other genetic regions for selection against ascites, but also for other complex traits. One problem with general selection against ascites is the tendency for smaller birds to be more resistant to the disease [32, 51], meaning strictly phenotype-based selection could negatively impact growth rate and feed efficiency. Given the specificity of the regions utilized in our MAS experiment, there is a strong potential that they could be easily integrated into the current breeding programs of poultry genetics companies. This could increase innate resistance to ascites without having to “back-track” over years of selection for growth traits. WGR and MAS hold great promise for targeted genetic selection in agricultural systems.

## Supplementary Information

**Additional file 1 Supplemental Table 1.** Feed formulation and composition used in both the floor and hypobaric chamber trials for the three feed phases.

## Data Availability

The datasets used and/or analyzed during the current study are available from the corresponding author on reasonable request.
