# Supplementary material for: SNP-based breeding for broiler resistance to ascites and evaluation of correlated production traits
Source: Hereditas. 2022 Jan 28;159:9. doi: 10.1186/s41065-022-00228-x (PMC8796538; doi:10.1186/s41065-022-00228-x)
Supplement: Supplementary file 1 — Additional file 1 Supplemental Table 1. Feed formulation and composition used in both the floor and hypobaric chamber trials for the three feed phases. [file 41065_2022_228_MOESM1_ESM.docx]

**Supplemental Table 1.** Feed formulation and composition used in both the floor and hypobaric chamber trials for the three feed phases.

| **Feed formulation** | | | | |
| --- | --- | --- | --- | --- |
| Ingredient name | Concentration | Starter | Finisher | Withdrawal |
| Corn, yellow | 7.5% | 55.0 | 61.9 | 67.6 |
| Soybean meal | 47% | 37.7 | 31.1 | 26.1 |
| Poultry fat |  | 3.4 | 3.0 | 2.7 |
| MonoDicalcium Phosphate |  | 1.43 | 1.37 | 1.31 |
| Fine limestone |  | 1.18 | 1.13 | 1.02 |
| Vitamin Premix |  | 0.05 | 0.05 | 0.05 |
| Mineral Premix |  | 0.20 | 0.20 | 0.20 |
| Sodium bicarbonate |  | 0.13 | 0.16 | 0.19 |
| Sodium chloride |  | 0.39 | 0.36 | 0.34 |
| Liquid-Methionine | 88% | 0.34 | 0.31 | 0.24 |
| L-Lysine HCl | 78% | 0.08 | 0.14 | 0.11 |
| Threonine | 98% | 0.09 | 0.09 | 0.05 |
| Choline | 70% | 0.10 | 0.10 | 0.08 |
| Total |  | 100.0 | 100.0 | 100.0 |
| **Final feed composition** | | | | |
| Nutrient | Unit |  |  |  |
| M.E. poultry | kcal/kg | 3010 | 3065 | 3113 |
| Protein, crude | % | 22.5 | 20.0 | 18.0 |
| Fat, crude | % | 5.1 | 4.9 | 4.7 |
| Calcium | % | 0.90 | 0.84 | 0.76 |
| Phosphorus, available | % | 0.45 | 0.42 | 0.38 |
| Sodium | % | 0.21 | 0.21 | 0.21 |
| Potassium | % | 1.04 | 0.93 | 0.83 |
| Chloride | % | 0.30 | 0.30 | 0.28 |
